# A novel all-fiber-based LiFePO_4_/Li_4_Ti_5_O_12_ battery with self-standing nanofiber membrane electrodes

**DOI:** 10.3762/bjnano.10.215

**Published:** 2019-11-13

**Authors:** Li-li Chen, Hua Yang, Mao-xiang Jing, Chong Han, Fei Chen, Xin-yu Hu, Wei-yong Yuan, Shan-shan Yao, Xiang-qian Shen

**Affiliations:** 1 Institute for Advanced Materials, School of Materials Science & Engineering, Jiangsu University, Zhenjiang 212013, China; 2 Institute of Clean Energy & Advanced Material, Southwest University, Chongqing, 400715, China

**Keywords:** 3D network, electrospinning, flexible electrodes, lithium ion battery, nanofiber, self-standing electrodes

## Abstract

Electrodes with high conductivity and flexibility are crucial to the development of flexible lithium-ion batteries. In this study, three-dimensional (3D) LiFePO_4_ and Li_4_Ti_5_O_12_ fiber membrane materials were prepared through electrospinning and directly used as self-standing electrodes for lithium-ion batteries. The structure and morphology of the fibers, and the electrochemical performance of the electrodes and the full battery were characterized. The results show that the LiFePO_4_ and Li_4_Ti_5_O_12_ fiber membrane electrodes exhibit good rate and cycle performance. In particular, the all-fiber-based gel-state battery composed of LiFePO_4_ and Li_4_Ti_5_O_12_ fiber membrane electrodes can be charged/discharged for 800 cycles at 1C with a retention capacity of more than 100 mAh·g^−1^ and a coulombic efficiency close to 100%. The good electrochemical performance is attributed to the high electronic and ionic conductivity provided by the 3D network structure of the self-standing electrodes. This design and preparation method for all-fiber-based lithium-ion batteries provides a novel strategy for the development of high-performance flexible batteries.

## Introduction

With the rapid development of renewable energy technologies, electric vehicles and electronic devices, energy storage technology has become a focus of global research [1–7]. The high demand for portable and flexible devices requires the development of efficient power supplies to maintain the normal operation of these devices [8–10]. Flexible lithium-ion batteries play a dominant role in the portable equipment market due to their high energy density, long life and environmental friendliness [11–14]. The electrode materials of conventional lithium-ion batteries (LIBs) are generally based on transition metal oxides containing lithium mixed evenly with conductive agents and adhesives. The electrode materials are then coated on metal current collectors [15–17]. However, the electrodes prepared by this method are easily separated from the collectors during repeated bending. Therefore, the design of electrodes that require no current collectors and are flexible to adapt to repeated bending and folding has become particularly important in the current LIB research. Researchers have found that two-dimensional (2D) or 3D free-standing electrode materials [18–21] can significantly improve the electrochemical performance while also offering light weight and superior mechanical properties [22–23].

LiFePO_4_ and Li_4_Ti_5_O_12_ have been widely developed and applied in LIBs for electric and hybrid vehicles due to their stable discharge potential, good cycling stability and environmental friendliness [24–26]. However, both of them have the disadvantages of low Li^+^ diffusion coefficient and poor electronic conductivity, which limit the practical applications in LIBs. Nanostructures of various shapes such as nanofibers [27–28], nanoparticles [29], nanotubes [30], nanowires [31], and nanosheets [32] can greatly shorten the conduction path of Li^+^, thus improving the Li^+^ conductivity. In addition, coating or blending with conductive carbon can significantly increase the electronic conductivity [33]. Nanofibers combining active substances with conductive carbon as flexible electrodes not only eliminate the need for current collector and binder, but also save the coating process. Moreover, due to the continuous fiber structure, the electronic conductivity of the material is increased, the utilization ratio of the active material is improved, and the structural stability of the material is enhanced [18,27–28]. Therefore, it is highly desirable to fabricate all-fiber-based batteries to achieve high performance for practical applications [34–35].

Electrospinning is an effective method to prepare long-range continuous nanofibers. By controlling the spinning and sintering process, nanofiber membrane materials can be easily formed with high porosity and stable structure, especially continuous conductive networks can be formed, which are very suitable for self-standing electrodes [18,21,28]. In recent years, an increasing number of reports on the preparation of fiber electrode materials by electrospinning were published [18,21,28,34–35].

**Figure 1:**
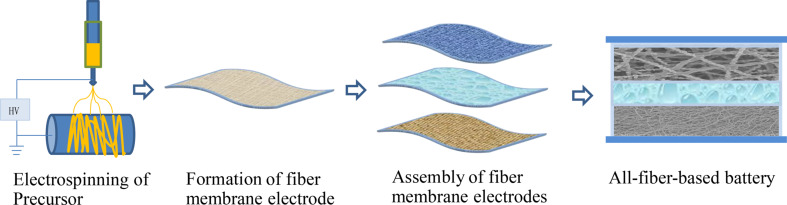
Fabrication process of all-fiber-based gel-state batteries.

In this paper, we describe the preparation of LiFePO_4_ and Li_4_Ti_5_O_12_ nanofiber membrane materials by a modified electrospinning method. We used the materials in all-fiber-based gel batteries (the battery fabrication process is schematically shown in Figure 1). The electrochemical properties of semi-batteries and full batteries were studied, and the mechanisms leading to the high performance of the batteries were subsequently investigated.

## Results and Discussion

### Morphology and phases of the nanofiber membranes

**Figure 2:**
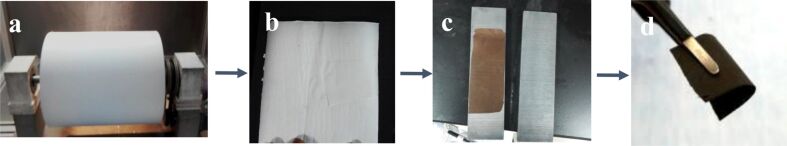
Digital photos of LiFePO_4_ nanofiber films. (a, b) The precursor fiber membrane; (c) the pre-treated fiber membrane; (d) the sintered fiber membrane.

Nanofiber membranes with high flexibility and stable structures can be successfully prepared by electrospinning and hot-pressing sintering as described in our previous works [36–39]. The specific experimental process for LiFePO_4_ and Li_5_Ti_4_O_12_ nanofiber membranes is shown in Figure 2. It can be seen that the sintered LiFePO_4_ nanofiber membrane keeps a stable structure and shows good bending ability. Particularly, adding polymers with different molecular weights to the precursors can adjust the distribution of grains in the fibers.

**Figure 3:**
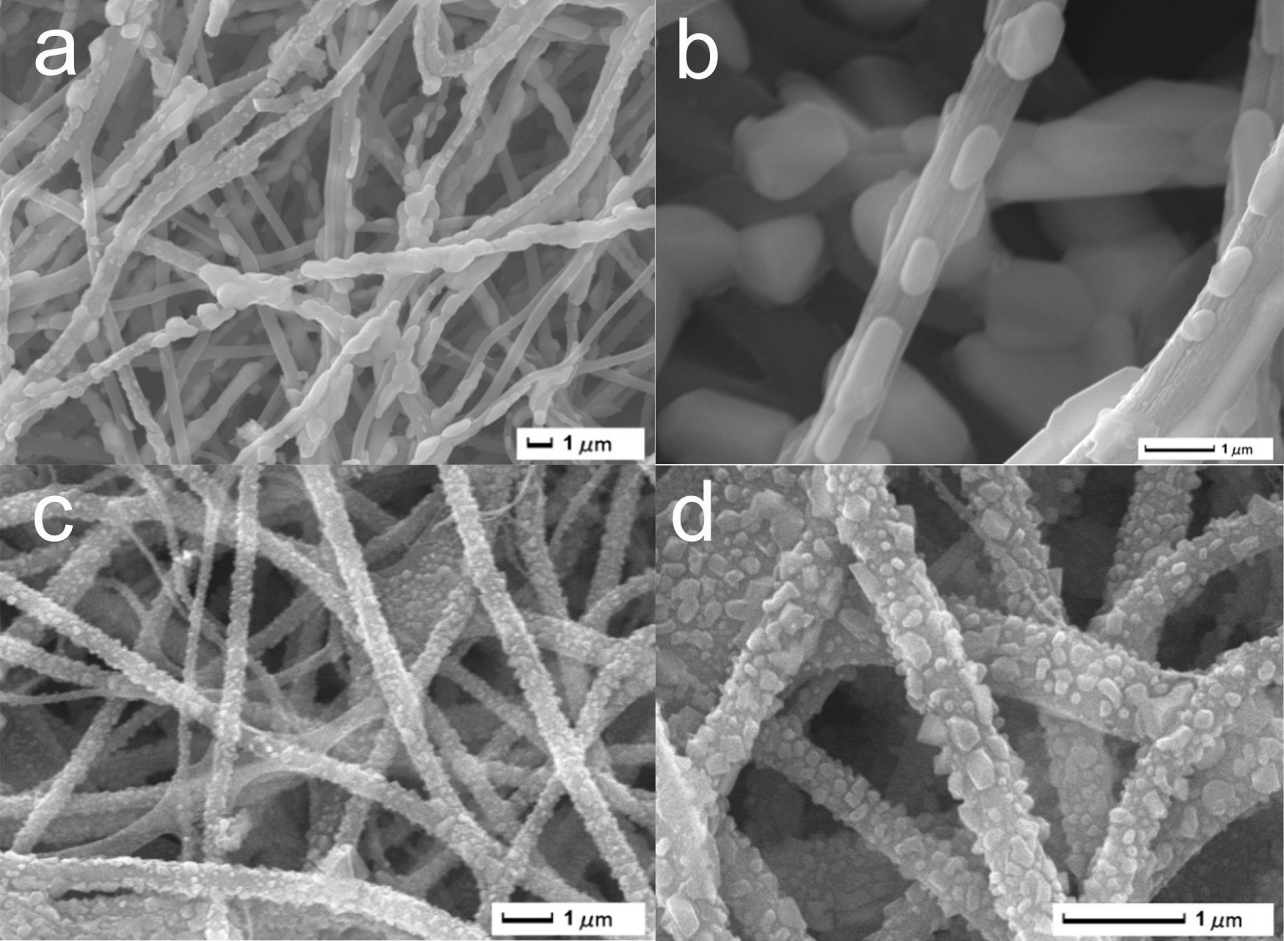
SEM pictures of LiFePO_4_ and Li_4_Ti_5_O_12_ nanofiber membranes. (a, b) LiFePO_4_; (c, d) Li_4_Ti_5_O_12_.

SEM images of LiFePO_4_ and Li_5_Ti_4_O_12_ nanofiber membranes are shown in Figure 3. It can be seen that the fiber membranes after heat treatment exhibit a 3D network structure, which is the reason for the high flexibility of the electrode. The high-magnification SEM images show uniform growth of crystal grains on the surface of the fibers for both LiFePO_4_ and Li_5_Ti_4_O_12_. The fiber diameter is less than 1 μm, and the grain size is between 200 and 300 nm. There are numerous channels between the fibers. This structure is beneficial for the penetration of electrolyte and the contact between active substances and electrolyte. The resistance of Li^+^ during charging and discharging of the battery decreases, and the internal structure of the material cannot collapse of deform easily. Thus, the structure of the material remains unchanged even after many cycles [40–41].

**Figure 4:**
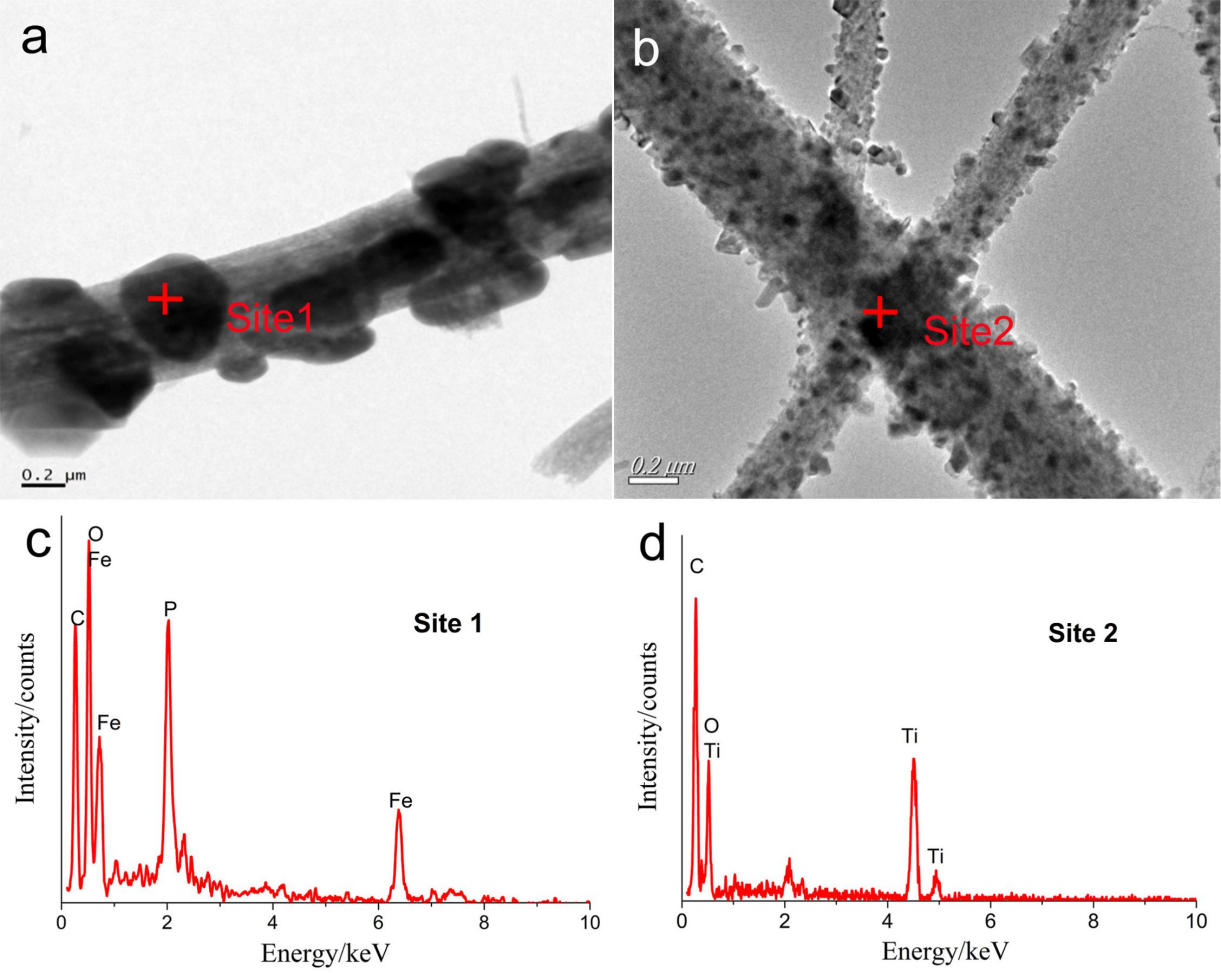
TEM pictures and EDS spectra of LiFePO_4_ and Li_4_Ti_5_O_12_ fibers. (a)TEM of a LiFePO_4_ fiber; (b) TEM of Li_4_Ti_5_O_12_ fibers; (c)EDS of LiFePO_4_ at site 1; (d) EDS of Li_4_Ti_5_O_12_ at site 2.

TEM images of the LiFePO_4_ and Li_5_Ti_4_O_12_ fibers in Figure 4 reveals that the active particles are uniformly distributed on the surface of the fibers, in agreement with the SEM images. EDS analysis shows either Fe, C, P, and O or Ti, C, and O on the fibers.

**Figure 5:**
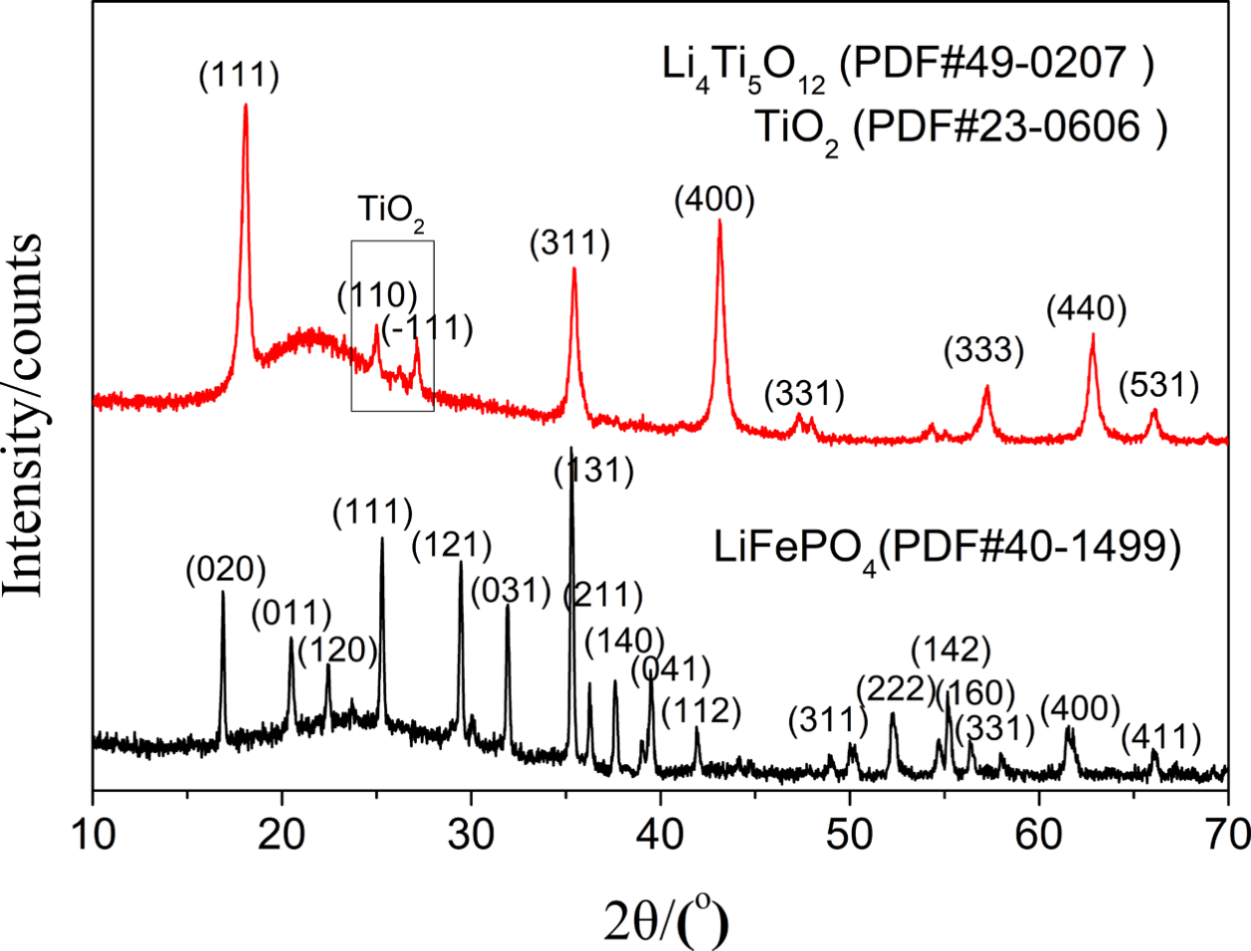
XRD patterns of LiFePO_4_ and Li_4_Ti_5_O_12_ fiber membranes.

**Figure 6:**
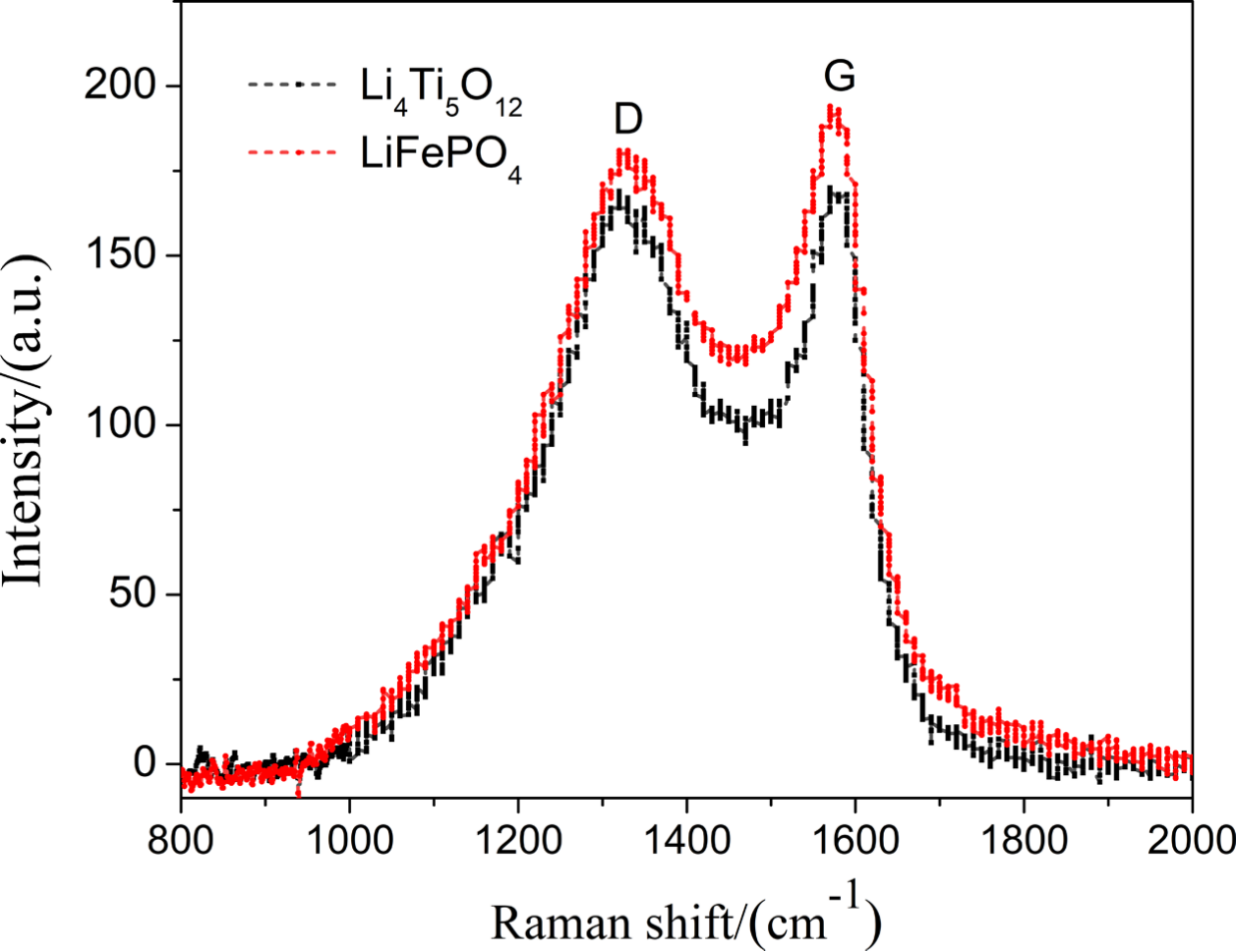
Raman spectra of LiFePO_4_ and Li_4_Ti_5_O_12_ fiber membranes.

The fibers were further examined using XRD and Raman spectroscopy. The XRD patterns (Figure 5) show that the diffraction peaks of sintered LiFePO_4_ and Li_4_Ti_5_O_12_ fibers coincide with those of the olivine LiFePO_4_ standard (PDF#40-1499) and the spinel Li_4_Ti_5_O_12_ standard (PDF#49-0207), respectively, which means that the sintered fibers contain the expected phases. Only a small amount of TiO_2_ was found in the diffraction peaks of Li_4_Ti_5_O_12_ fibers, which may be related to the sintering atmosphere of Li_4_Ti_5_O_12_ fibers. Because the sintering of Li_4_Ti_5_O_12_ fibers was carried out under the pressure of a graphite plate in N_2_ atmosphere, the sintering atmosphere is a partially reductive inert atmosphere. However, TiO_2_ itself is a relatively stable anode material [42], so the appearance of such impurities would not affect the performance of the electrode. The Raman spectra of the two fiber materials in Figure 6 show two characteristic peaks at 1350 cm^−1^ and 1580 cm^−1^ corresponding to the D and the G band of carbon, respectively. The ratio of the two peaks reflects the degree of graphitization. This kind of composite not only benefits the flexibility of the fibers, but also grants good conductivity to the fibers, which is crucial for the preparation of free-standing electrodes.

### Electrode performance

**Figure 7:**
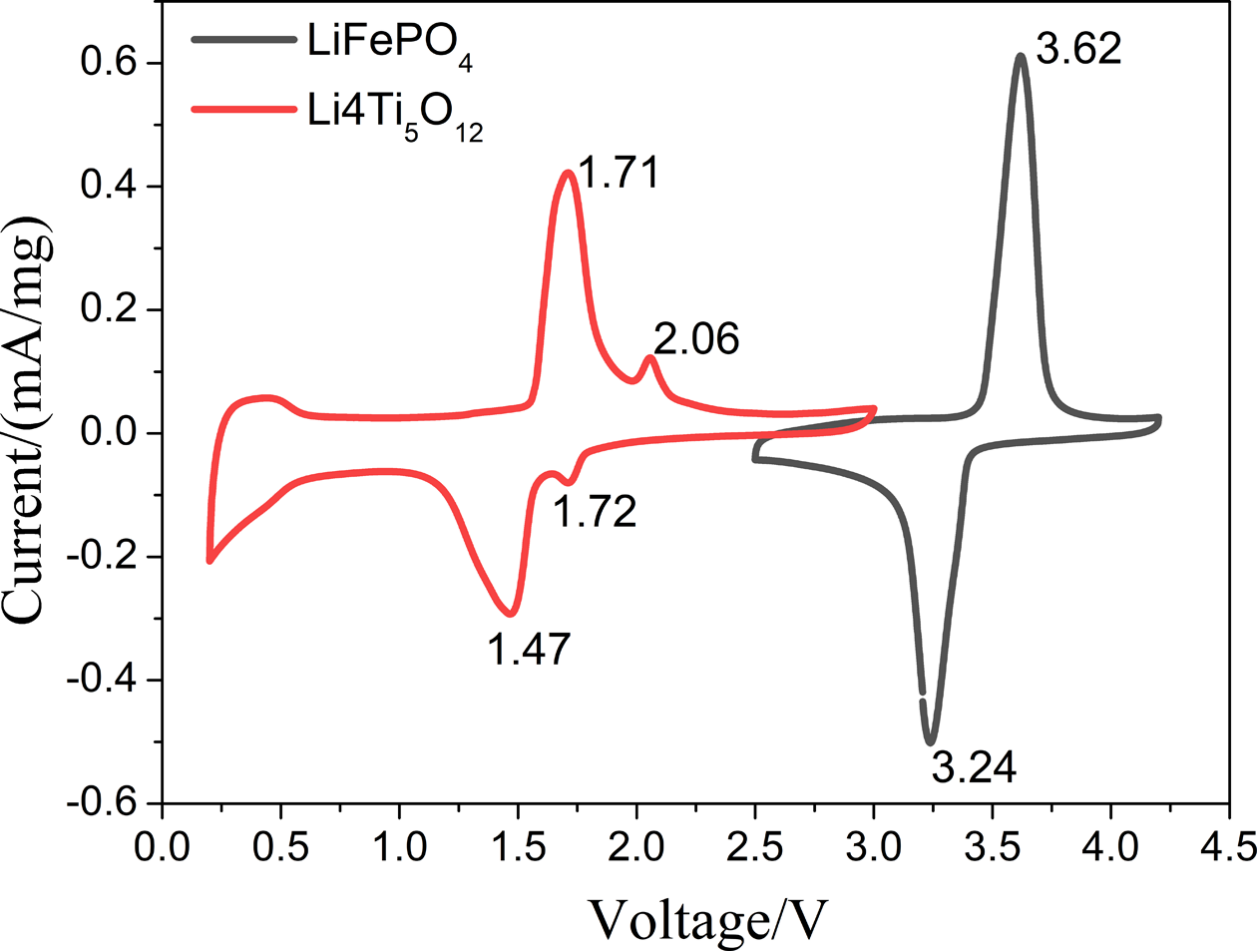
CV curves of LiFePO_4_ and Li_4_Ti_5_O_12_ fiber membrane electrodes.

The two fiber membranes were directly cut into free-standing electrodes, and coin-cell batteries were assembled for a series of performance tests. Figure 7 are the cyclic voltammetry curves (CV, the second cycle) of the two electrodes. It can be seen that the strong redox peaks of the LiFePO_4_ cathode appear at 3.62 V and 3.24 V, respectively. This corresponds to the Li^+^ removal from and intercalation in LiFePO_4_, i.e., the redox process of Fe^3+^/Fe^2+^ [24]. The redox peaks of Li_4_Ti_5_O_12_ at 1.71 V and 1.47 V correspond to the Li^+^ removal from and intercalation in Li_4_Ti_5_O_12_, i.e., the redox process of Ti^4+^/Ti^3+^ [25]. The two small redox peaks at 2.06 V and 1.72 V correspond to the Li^+^ removal from and intercalation in TiO_2_.

**Figure 8:**
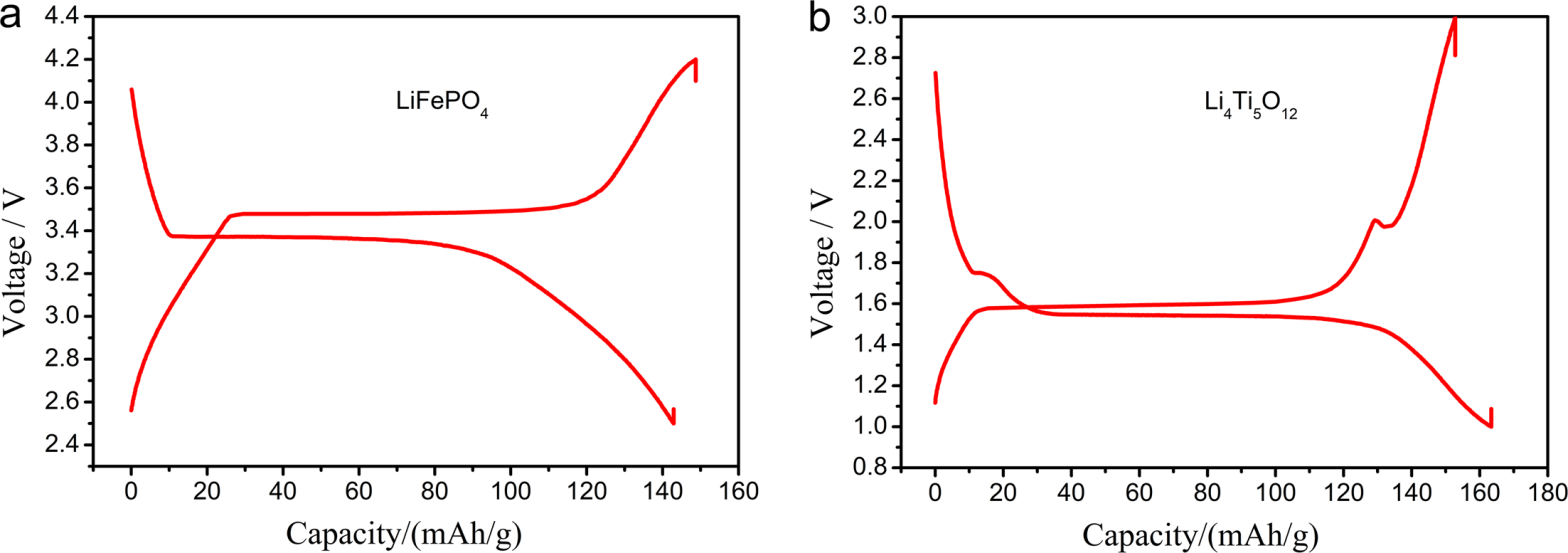
Charge–discharge curves of LiFePO_4_ and Li_4_Ti_5_O_12_ fiber membrane electrodes.

From the charge–discharge curves in Figure 8 (the second cycle), it can be seen that both electrodes have obvious charge–discharge plateaus at about 3.5 V and 1.5 V, which are consistent with the CV curves, corresponding to the processes of Li^+^ removal from and intercalation in LiFePO_4_ and Li_4_Ti_5_O_12_, respectively. It is noteworthy that two smaller plateaus for charging and discharging of TiO_2_ are also found on the charge-discharge curves of Li_4_Ti_5_O_12_, which is in agreement with the redox peaks in the CV curves.

**Figure 9:**
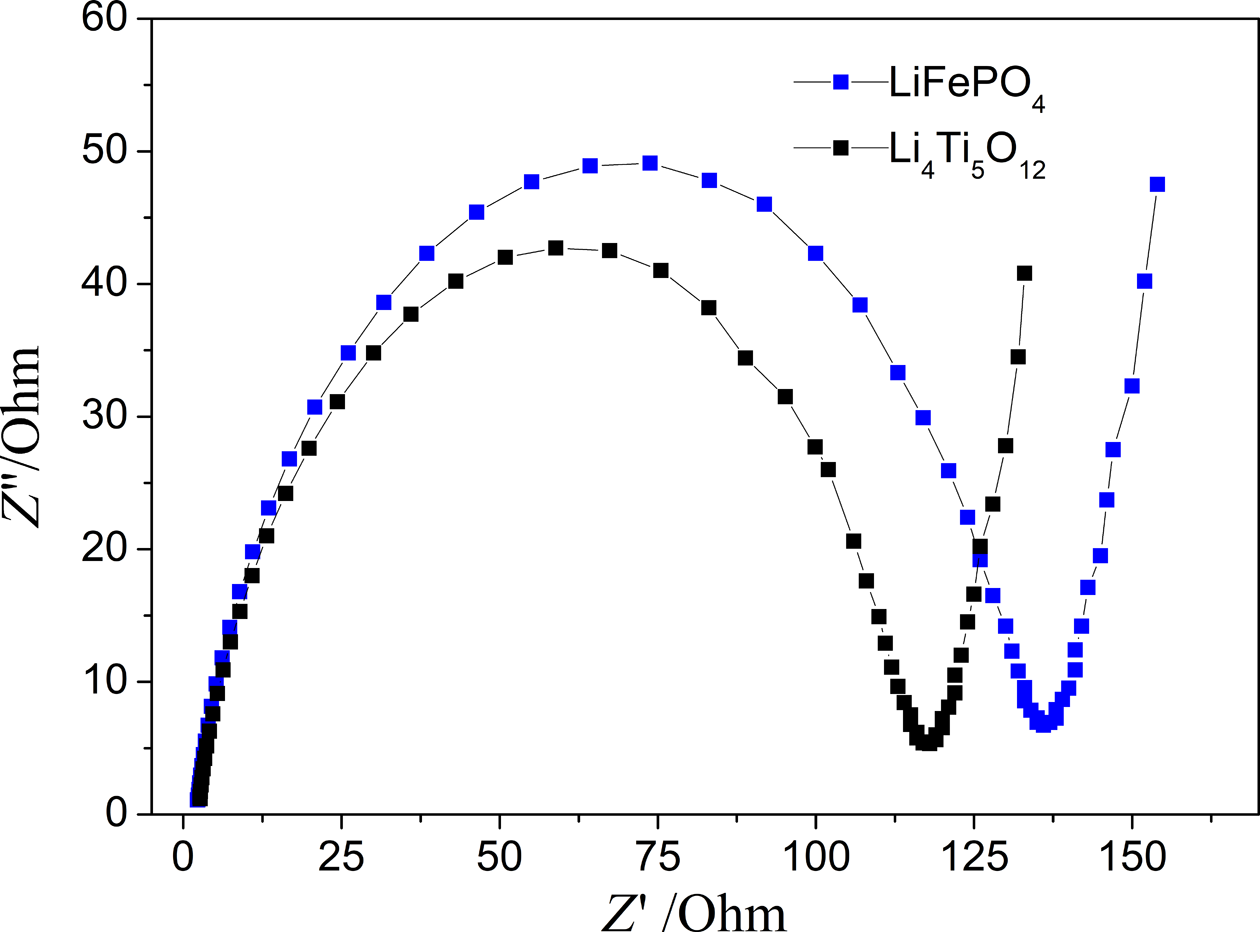
EIS curves of LiFePO_4_ and Li_4_Ti_5_O_12_ fiber membrane electrodes.

Figure 9 shows the electrical impedance spectroscopy curves of the two kinds of fiber membrane electrodes. It can be seen that there is a regular semicircle in the high-frequency region, which represents the magnitude of the charge transfer resistance *R*_ct_. The oblique line in the low-frequency region is larger than 45°, which is the impedance of the Li^+^ diffusion process in the electrode. Overall, although the electrodes did not use metal collectors, both electrodes showed a smaller charge transfer impedance and a smaller ion transfer impedance, which illustrates the effect of the 3D conductive network and the high porosity on electron and ion transport [40–41].

**Figure 10:**
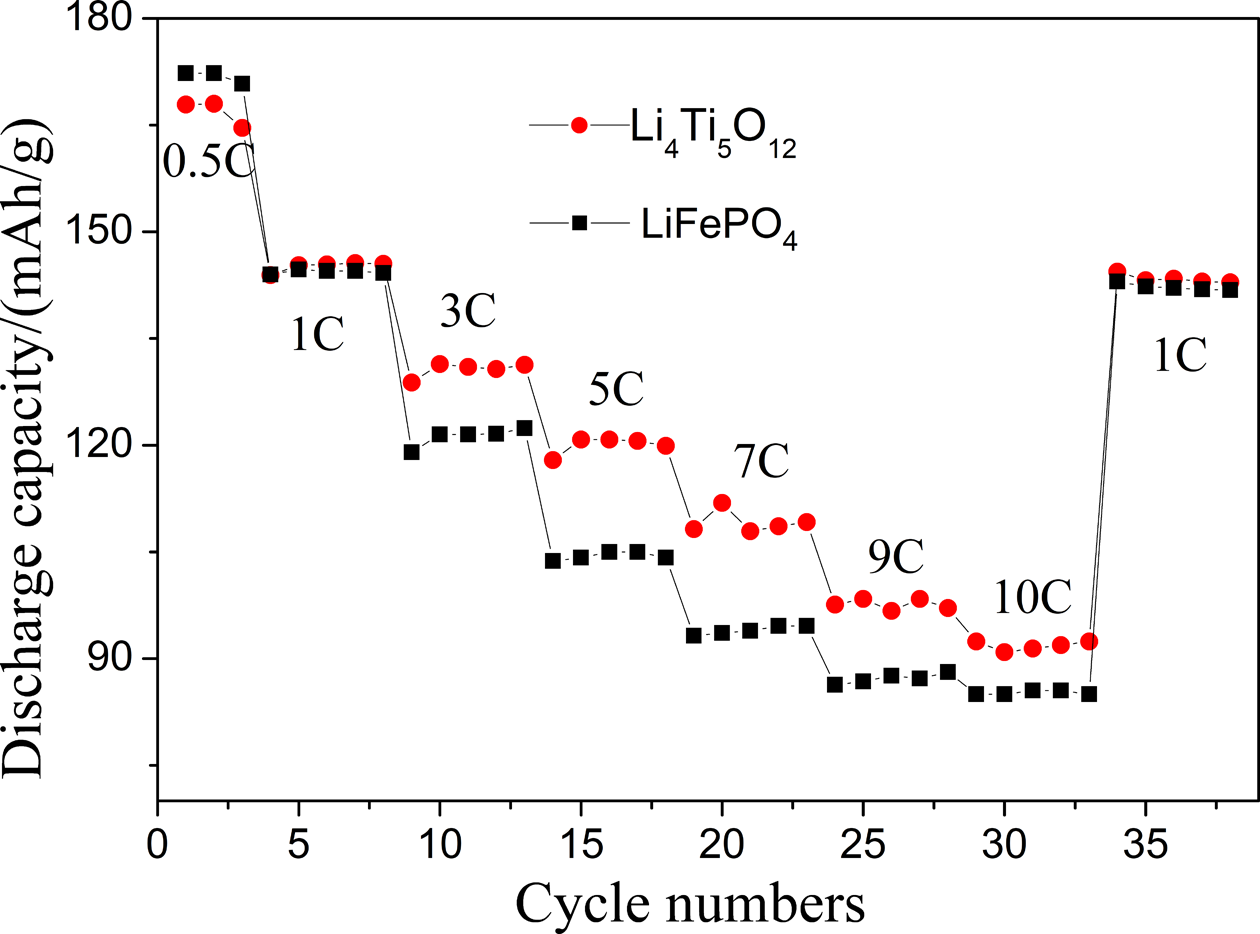
Rate performance of LiFePO_4_ and Li_4_Ti_5_O_12_ fiber membrane electrodes.

Figure 10 shows the rate performance of LiFePO_4_ and Li_4_Ti_5_O_12_ fiber membrane electrodes. Both electrodes can be charged and discharged normally from 0.5C to 10C. When the current density returns to 1C, the discharge capacity goes back to the initial value, indicating that the electrodes have a good reversibility. It is noteworthy that with the increase of current density, the capacity difference between LiFePO_4_ and Li_4_Ti_5_O_12_ gradually widens, which may be related to the different grain sizes of active substances in these two electrodes. It is known that nanomerization could improve the rate capability and insertion kinetics of electrode materials. The SEM and TEM images in Figure 3 and Figure 4 show that the grain size of LiFePO_4_ is larger than that of Li_4_Ti_5_O_12_, which is the possible reason why the capacity of the LiFePO_4_ electrode decreases more quickly with the increase of rate.

**Figure 11:**
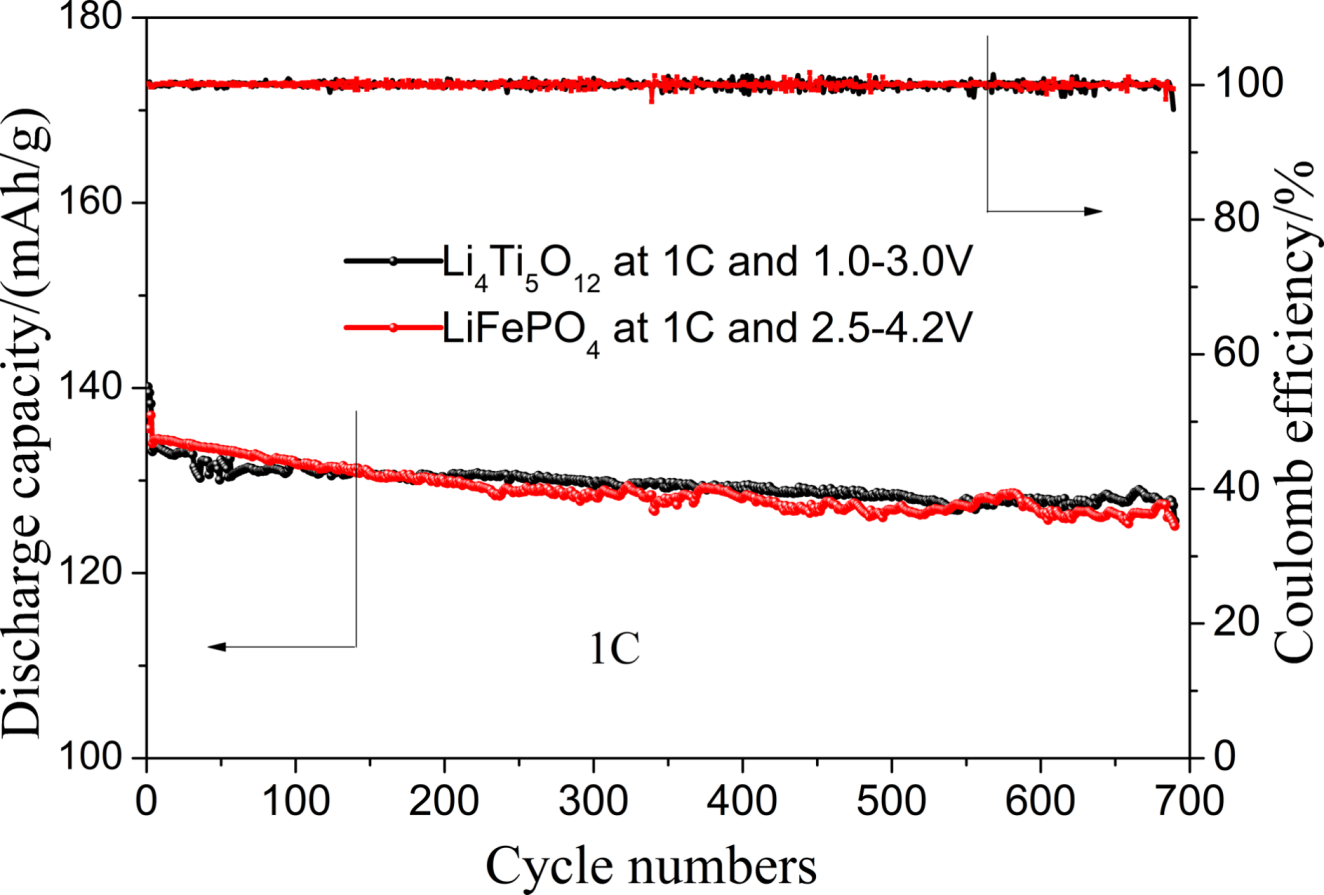
Cycle performance of LiFePO_4_ and Li_4_Ti_5_O_12_ fiber membrane electrodes.

The cycle performance of the electrodes at 1C was further tested. Figure 11 shows the charge–discharge cycle performance of the two electrodes at 1C. It can be seen that both electrodes can be stably cycled for more than 700 cycles, and the discharge capacity decreases from the initial 135 to 125 mAh·g^−1^, showing a good stability. Meanwhile, the two kinds of electrodes also show a high coulombic efficiency close to 100%. It further shows that the free-standing electrodes have excellent cycling stability and high charge–discharge reversibility.

### Battery performance

**Figure 12:**
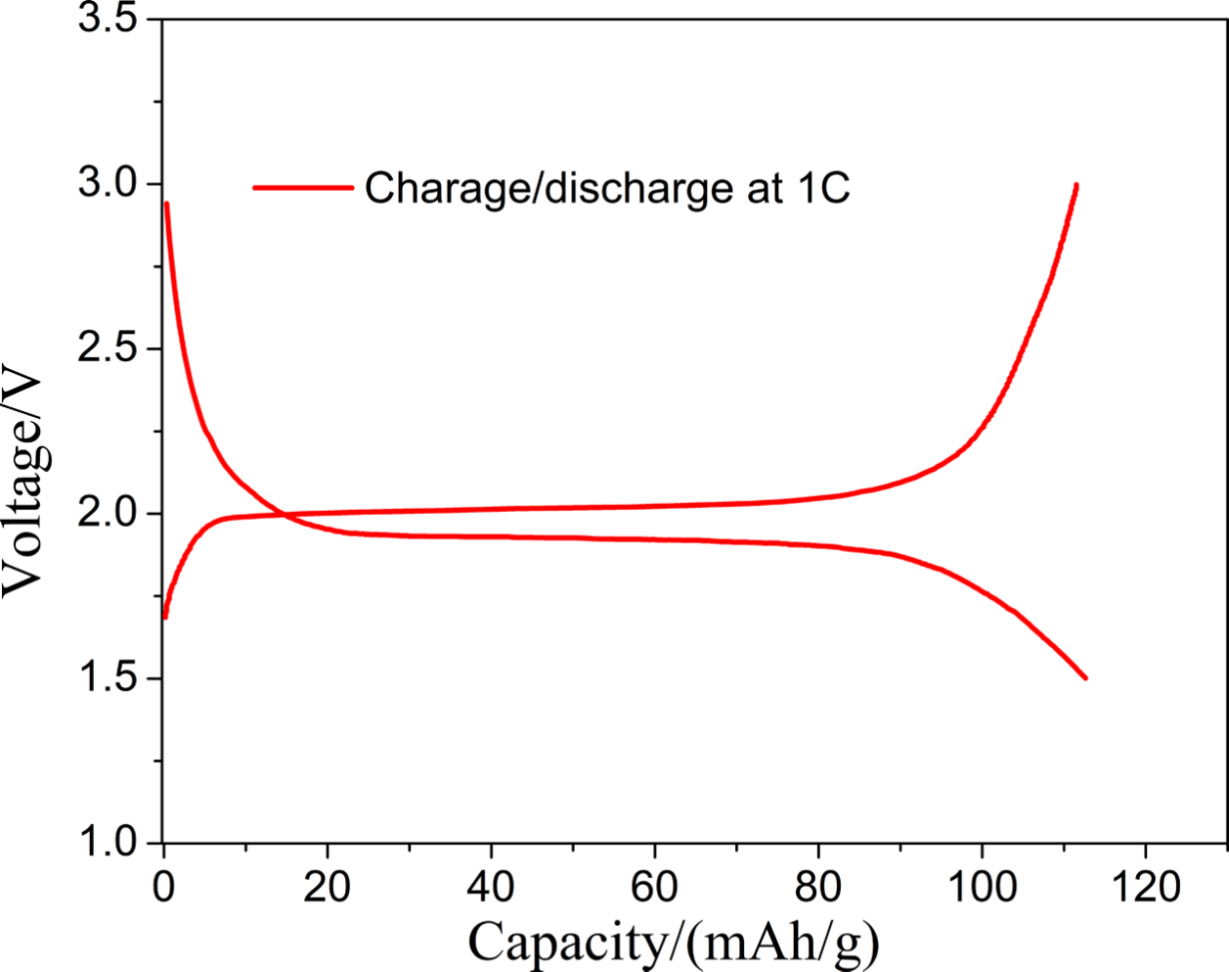
Charge–discharge curves of the LiFePO_4_//Li_4_Ti_5_O_12_ all-fiber battery at 1C.

The two free-standing electrodes were assembled to a full battery with the prepared electrolyte membrane (Figure S1, Supporting Information File 1), and the rate and the cycling performance of the battery were tested. From the charge–discharge curve in Figure 12, it can be seen that the LiFePO_4_//Li_4_Ti_5_O_12_ battery has a very flat charge–discharge plateau in the voltage window of 1.5–3.0 V, similar to the conventional LiFePO_4_//Li_4_Ti_5_O_12_ battery. It shows that LiFePO_4_ and Li_4_Ti_5_O_12_ fiber membrane electrodes match and can deliver their respective capacities.

**Figure 13:**
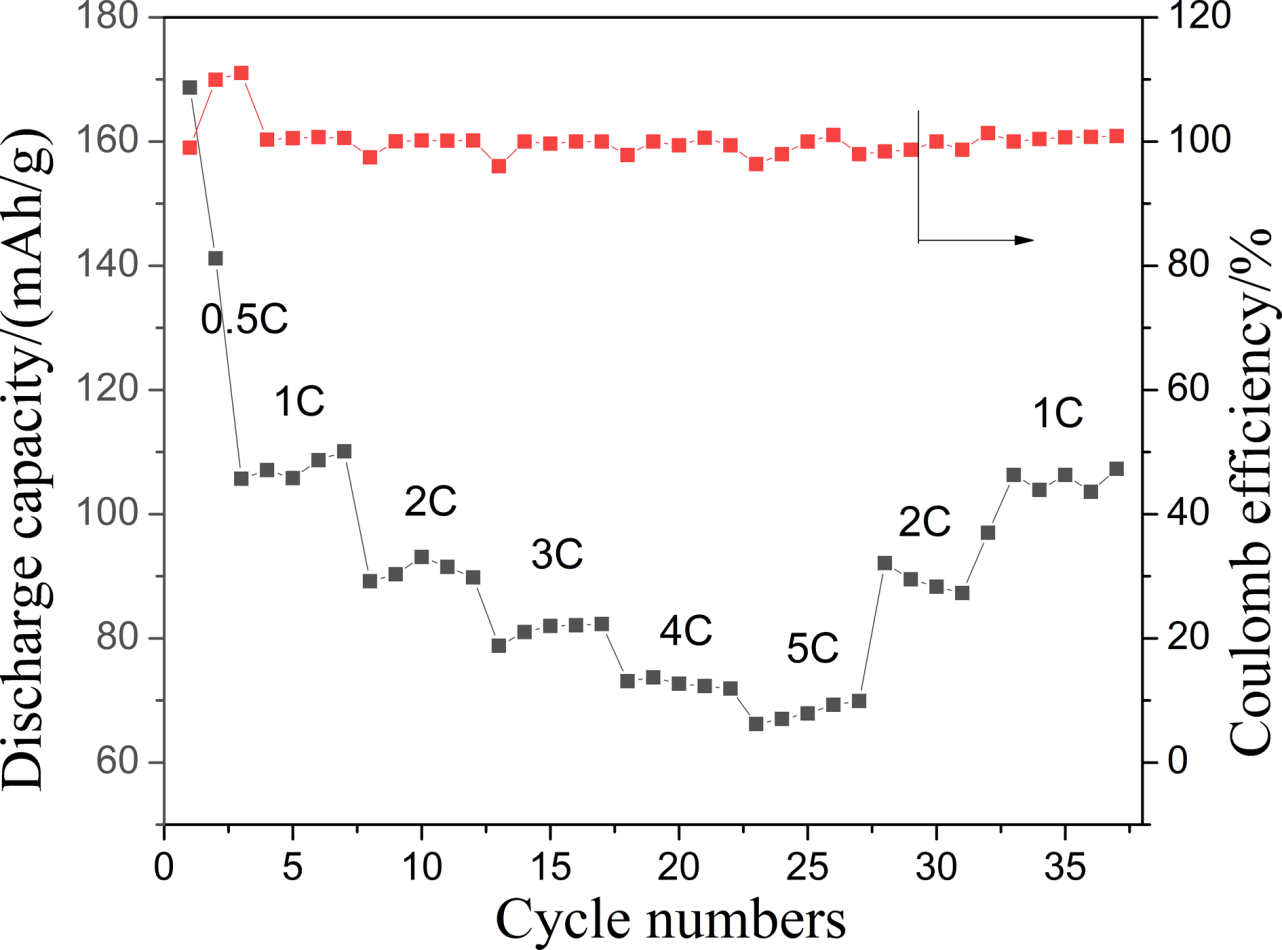
Rate performance of the LiFePO_4_//Li_4_Ti_5_O_12_ all-fiber battery.

**Figure 14:**
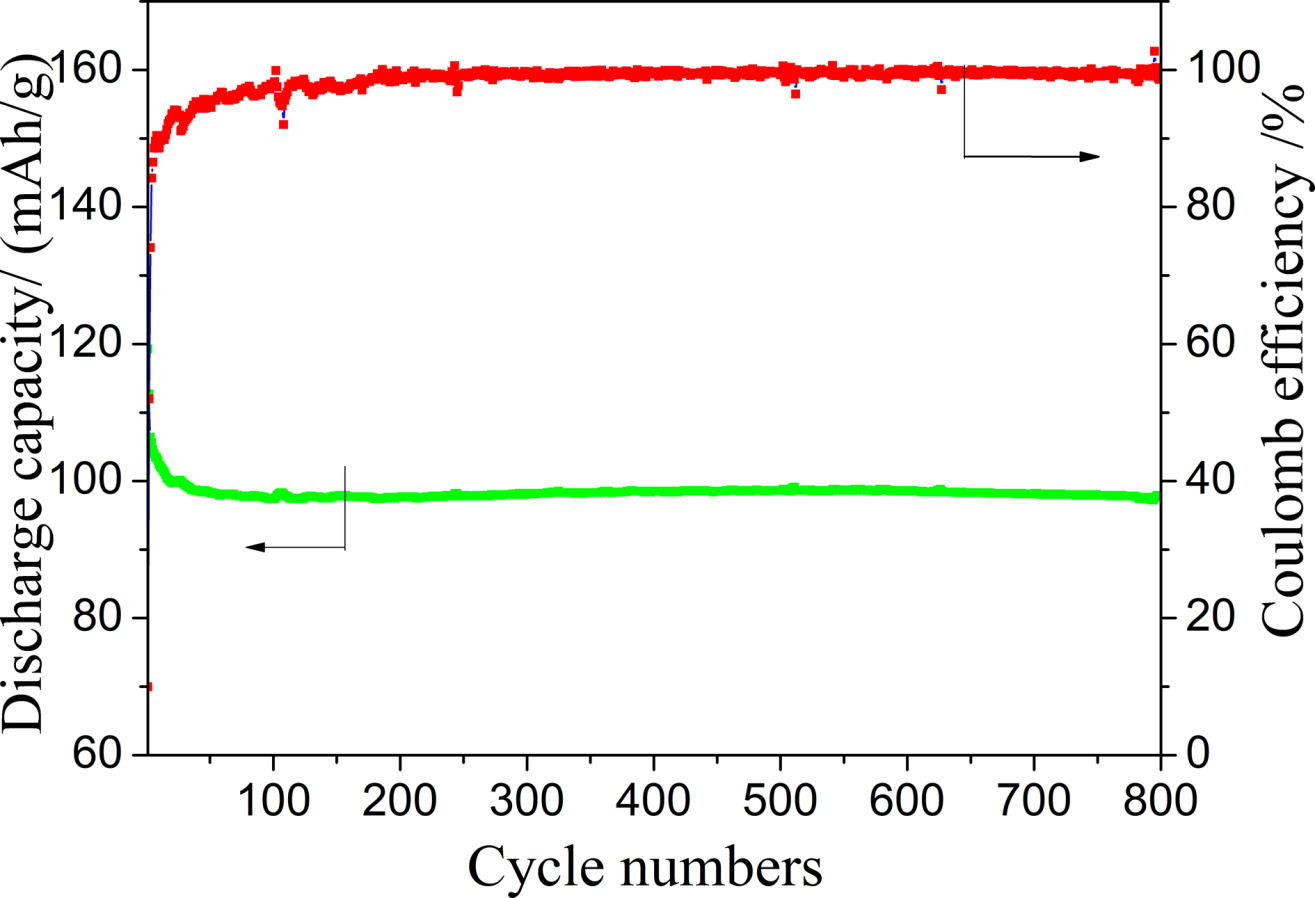
Cycle performance of the LiFePO_4_//Li_4_Ti_5_O_12_ all-fiber battery at 1C.

Figure 13 shows the rate performance of the LiFePO_4_//Li_4_Ti_5_O_12_ battery. The battery can be normally charged and discharged from 0.5C to 10C. The specific discharge capacity at 1C is close to 110 mAh·g^−1^, and reaches about 70 mAh·g^−1^ at 5C. When the rate is set to 1C again, the specific capacity is restored to the initial state. The cycling performance of the battery was also tested. As shown in Figure 14, the battery was continuously cycled up to 800 cycles at 1C, and the remaining capacity was over 100 mAh·g^−1^. The coulombic efficiency is close to 100% except for the first few cycles.

**Figure 15:**
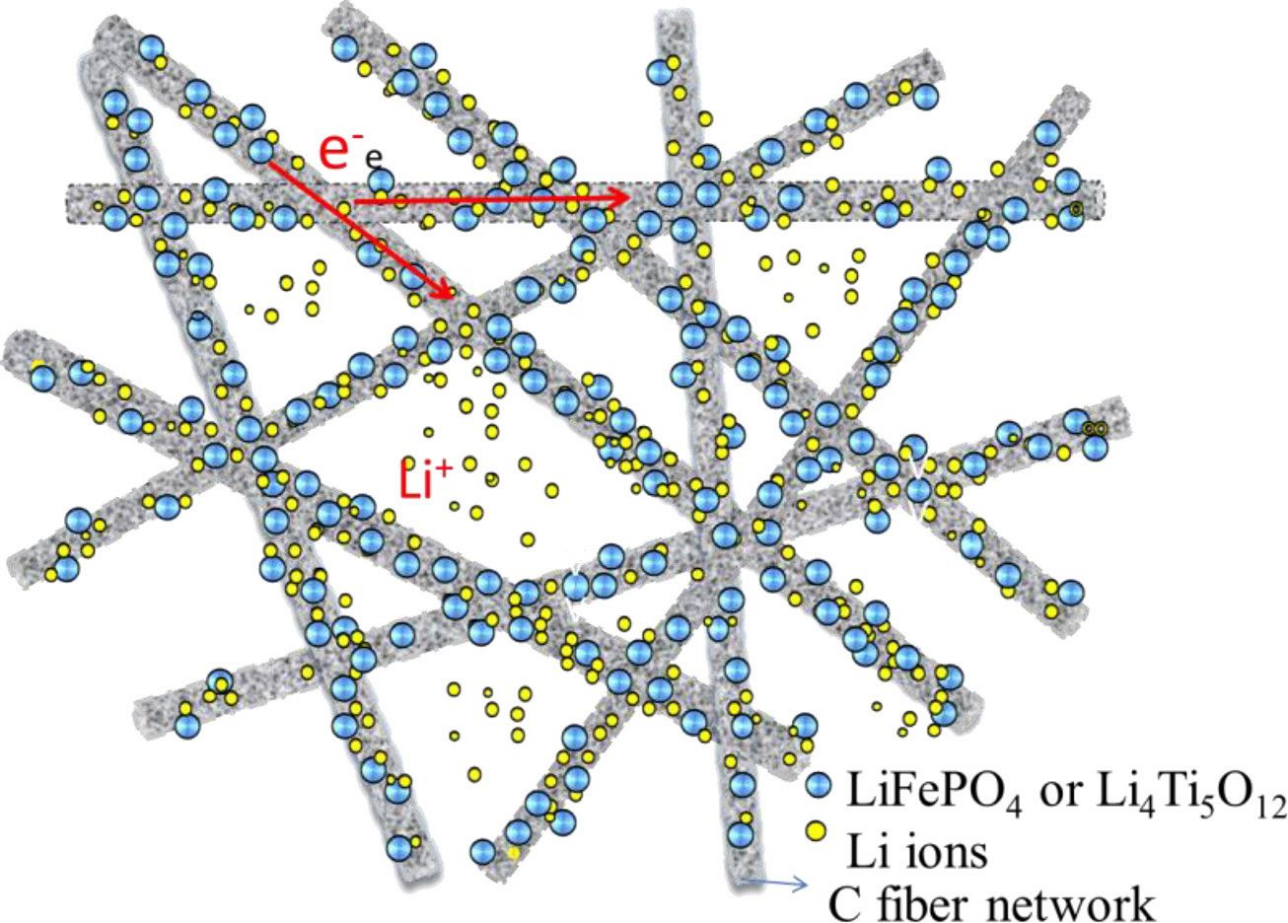
Sketch of 3D network structure for fast electron and ion transport.

Table S1 in Supporting Information File 1 lists the electrochemical performance obtained in some related works. It can be seen that the flexible self-standing LiFePO_4_/C fiber membrane cathode and Li_4_Ti_5_O_12_/C fiber membrane anode in this work show a comparable electrochemical performance. In addition, this all-fiber-based LiFePO_4_//Li_4_Ti_5_O_12_ full battery can be cycled at 1C for 800 times. Figure S2 (Supporting Information File 1) shows a photo and SEM pictures of the LiFePO_4_ and Li_4_Ti_5_O_12_ fiber membrane electrodes after the charge–discharge cycles. It can be seen that the composite electrodes still keep the 3D network structure after many cycles. TO summarize, the high rate and good cycling performance are mainly attributed to the high electronic and ionic conductivity of the free-standing electrodes with a stable three-dimensional network structure as shown in Figure 15, in which the high porosity, stable structure, and the continuous conductive networks provide the electrodes with fast electronic and ionic transport paths [22–23,34–35]. This design and fabrication of all-fiber-based batteries provides a novel strategy for the development of advanced flexible lithium-ion batteries.

## Conclusion

LiFePO_4_ and Li_4_Ti_5_O_12_ nanofiber membrane materials were successfully obtained by electrospinning and subsequent heat treatment. The nanofiber membranes were directly used as free-standing electrodes in LIBs and showed superior electrochemical performance. The battery consisting of these two electrodes can be charged and discharged for 800 cycles at 1C with a remaining capacity of 100 mAh·g^−1^ and a coulombic efficiency close to 100%. The excellent performance is attributed to the high electronic and ionic conductivity of the free-standing electrodes with 3D network structure. This design and fabrication of all-fiber-based LIBs shows a great potential in the development of flexible LIBs.

## Experimental

### Preparation of nanofiber membrane electrodes

LiFePO_4_ and Li_4_Ti_5_O_12_ nanofiber membranes were prepared via a modified electrospinning method. The typical preparation processes were as follows:

**LiFePO_4_:** 0.007 mol of Fe(NO_3_)_3_·9H_2_O and C_2_H_3_O_2_Li·2H_2_O, H_3_PO_4_ were dissolved in 29 g of *N*,*N*-dimethylformamide (DMF) to obtain solution A; 4 g of polyacrylonitrile (PAN) and 2 g of polyvinylpyrrolidone (PVP) were dissolved in 29 g of DMF to obtain solution B. A precursor spinning solution for the LiFePO_4_ nanofiber membrane is obtained after mixing A and B solutions. From the precursor solution a precursor fiber membrane was formed under a voltage of 25 kV, which was followed by a pre-oxidation at 260 °C for 2 h and then calcination at 800 °C for 10 h in N_2_ atmosphere (Figure 2c,d).

**Li_4_Ti_5_O_12_:** 0.01 mol tetra-*n*-butyl titanate (C_16_H_36_O_4_Ti), 0.9593 g C_2_H_3_O_2_Li·2H_2_O, and 0.25 mL HNO_3_ were dissolved in 29 g of *N*,*N*-dimethylformamide (DMF) to obtain solution A; 4 g of polyacrylonitrile (PAN) and 2 g of polyvinylpyrrolidone (PVP) were dissolved in 29 g of DMF to obtain solution B. A precursor spinning solution for the Li_4_Ti_5_O_12_ nanofiber membrane was obtained after mixing A and B solutions. From the precursor solution a precursor fiber membrane was formed under a voltage of 25 kV, followed by a pre-oxidation at 260 °C for 2 h and then calcination at 800 °C for 5 h in N_2_ atmosphere.

### General characterization

A Rigaku D/Max2500X-ray diffractometer was used to determine the crystalline structure of the composite fibers. A JSM-5600LV scanning electron microscope and a JEOL JEM2010 transmission electron microscope were used to observe the morphology and microstructures of the fibers.

The electrochemical performance of the electrodes was evaluated in assembled CR2025 coin cells. The nanofiber membranes were directly cut into self-standing electrodes with a diameter of 12 mm. The carbon content in the fiber membrane electrodes is 25–27%, and the active mass of one piece of electrode is 2.5–3.0 mg·cm^−2^. The coin cells were assembled in an Ar-filled glovebox by using lithium foil as anode and 1 M LiPF_6_ electrolyte (ethylene carbonate/dimethyl carbonate/ethyl methyl carbonate = 1:1:1). A Celgard 2400 polypropylene membrane was used as separator. The cyclic voltammetry and electrochemical impedance spectroscopy measurements were carried out using a VMP2 electrochemical workstation. EIS was measured in the frequency range of 0.1 Hz to 100 kHz, with a disturbance amplitude of 10 mV. The galvanostatic cycle tests were carried out at different current densities.

### Assembly of LFP//LTO all-fibers-based battery

Polyvinylidene fluoride hexafluoropropylene (PVDF-HFP) copolymer was used as the electrolyte membrane matrix in this experiment, and acetone was used as the solvent. The PVDF-HFP solution was prepared by dissolving PVDF-HFP in acetone (PVDF-HFP (mass/g): acetone (volume/mL) = 1:15) through stirring at room temperature. The solution was transferred to a PTFE dish, and dried naturally until the solvent evaporates completely. The obtained electrolyte membrane is shown in Figure S1a (Supporting Information File 1). The optimum thickness of the electrolyte membrane is 60–70 μm.

The prepared electrolyte membrane was then punched into a disk with a diameter of 18 mm (Figure S1b, Supporting Information File 1). Before the assembly of the battery, the cut membrane was first soaked in LiPF_6_ electrolyte for 2–3 h. A gel-state 2025 type coin-cell battery was then assembled in an Ar-filled glovebox using this membrane as the gel electrolyte, and the prepared LiFePO_4_ and Li_4_Ti_5_O_12_ nanofiber membranes as cathode and anode respectively. This battery was subsequently subjected to rate and cycling tests.

## Supporting Information

A comparison between this work with related literature references, and photographs and SEM pictures of the LiFePO_4_ and Li_4_Ti_5_O_12_ fiber membrane electrodes after 800 cycles of the battery.

File 1 Additional experimental data.
