# Supplementary material for: A novel all-fiber-based LiFePO4/Li4Ti5O12 battery with self-standing nanofiber membrane electrodes
Source: Beilstein J Nanotechnol. 2019 Nov 13;10:2229–37. doi: 10.3762/bjnano.10.215 (PMC6880811; doi:10.3762/bjnano.10.215)
Supplement: File 1 — Additional experimental data. [file Beilstein_J_Nanotechnol-10-2229-s001.pdf]

BEILSTEIN JOURNAL OF NANOTECHNOLOGY

Supporting Information

for

A novel all-fiber-based $LiFePO_4/Li_4Ti_5O_{12}$ battery with self-standing nanofiber membrane electrodes

Li-li Chen, Hua Yang, Mao-xiang Jing, Chong Han, Fei Chen, Xin-yu Hu, Wei-yong Yuan, Shan-shan Yao and Xiang-qian Shen

Beilstein J. Nanotechnol. **2019,** *10,* 2229–2237. doi:10.3762/bjnano.10.215

Additional experimental data

License and Terms: This is a supporting information file under the terms of the Creative Commons Attribution License (http://creativecommons.org/licenses/by/4.0). Please note that the reuse, redistribution and reproduction in particular requires that the authors and source are credited.
The license is subject to the *Beilstein Journal of Nanotechnology* terms and conditions: (https://www.beilstein-journals.org/bjnano)

The obtained electrolyte membrane is shown in Figure S1a. The optimum thickness of the electrolyte membrane is 60–70 μm. The prepared electrolyte membrane was then punched into a disk with a diameter of 18 mm (Figure S1b).

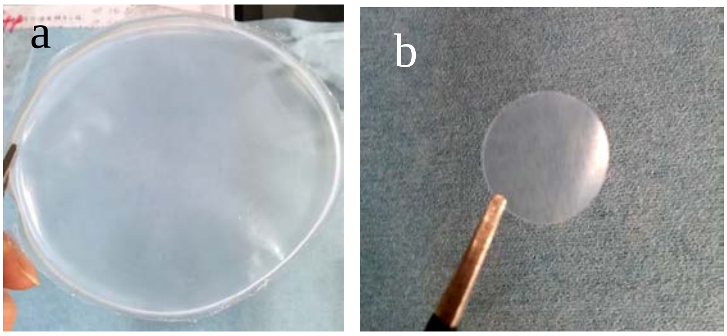

Figure S1: Pictures of the gel electrolyte.

Table S1 lists the electrochemical performance achieved in some related works. It can be seen that the flexible self-standing $LiFePO_4$/C fiber membrane cathode and $Li_4Ti_5O_{12}$/C fiber membrane anode in this work show a comparable electrochemical performance. In addition, the full all-fiber-based $LiFePO_4/Li_4Ti_5O_{12}$ battery can be cycled at 1C for 800 times.

Table S1: Comparison between this work and related references.

No.	components	method	performance	reference
**1**	$LiFePO_4$/C short fibers	electrospinning	160 mAh/g at 0.06C for 10 cycles	*ACS Appl. Mater. Interf.* **2010,** *2,* 212–218. doi:10.1021/am900656y
**2**	$LiFePO_4$ powder and $LiFePO_4$ nanofibers	solid-state blending	160 mAh/g at 0.1C for 40 cycles	*Ceram. Int.* **2015,** *41,* 1963–1969. doi:10.1016/j.ceramint.2014.08.120
**3**	$Li_4Ti_5O_{12}$/C short fibers	hydrothermal and ion-exchange	109 mAh/g at 20C for 50 cycles	*J. Alloys Compd.* **2017,** *721,* 545–553. doi:10.1016/j.jallcom.2017.06.035
**4**	carbon-coated $Li_4Ti_5O_{12}$ porous fibers	sol–gel	135 mAh/g at 5C for 100 cycles	*Electrochim. Acta* **2018,** *283,* 1418–1424. doi:10.1016/j.electacta.2018.07.127
**5**	$Li_3V_2(PO_4)_3$/C short fibers	electrospinning	103 mAh/g at 20C for 120 cycles	*Solid State Ionics* **2017,** *305,*36–42. 10.1016/j.ssi.2017.04.019
**6**	$LiMn_2O_4$ fibers|elled PVdF-HFP|$TiNb_2O_7$ fibers	electrospinning	116 mAh/g at 150 mA/g	*ACS Appl. Mater Interfaces* **2014,** *6,* 8660–8666. doi:10.1021/am501464d
**7**	$LiFePO_4$/C fiber membrane<br>$Li_4Ti_5O_{12}$/C fiber membrane<br>$LiFePO_4$/C fiber membrane|gelled PVdF|$Li_4Ti_5O_{12}$/C fiber membrane	electrospinning	135 mAh/g at 1C for 700 cycles<br>100mAh/g at 1C for 800 cycles	this work

Figure S2 shows a photograph and SEM pictures of the $LiFePO_4$ and $Li_4Ti_5O_{12}$ fiber membrane electrodes after charge–discharge cycles. It can be seen that the composite electrodes still keep the 3D network structure after many cycles. This indicates that these fiber membranes possess a high structural stability during charge–discharge cycles.

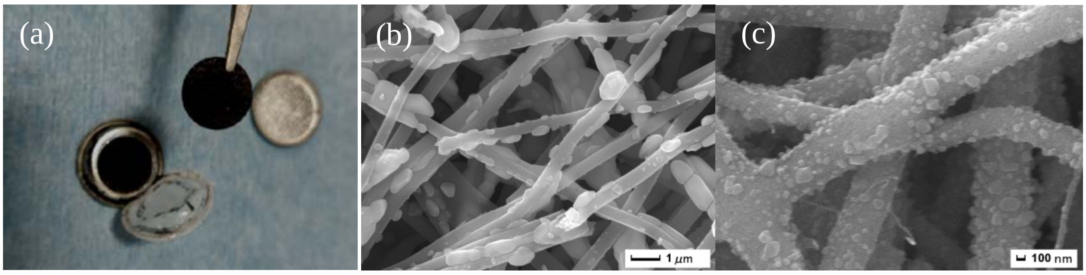

Figure S2: (a) Photograph and SEM pictures of (b) the $LiFePO_4$ and (c) the $Li_4Ti_5O_{12}$ fiber membrane electrode after 800 cycles.
